# Global Stability of Predator-Prey System with Alternative Prey

**DOI:** 10.5402/2013/898039

**Published:** 2012-08-08

**Authors:** Banshidhar Sahoo

**Affiliations:** Department of Mathematics, Daharpur A.P.K.B Vidyabhaban, Paschim Medinipur, West Bengal, India

## Abstract

A predator-prey model in presence of alternative prey is proposed. Existence and local stability conditions for interior equilibrium points are derived. Global stability conditions for interior equilibrium points are also found. Bifurcation analysis is done with respect to predator's searching rate and handling time. Bifurcation analysis confirms the existence of global stability in presence of alternative prey.

## 1. Introduction

**Figure 1:**
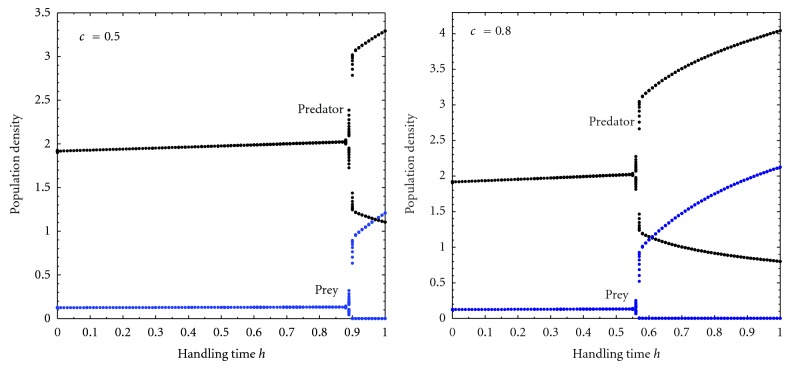
Bifurcation diagram of the prey and predator species with respect to handling time *h* for *k* = 3, *a* = 0.5, *b* = 0.2, and *d* = 0.025.

The classical predator-prey model based on the logistic growth principle and Hollings predation theory is as follows:

(1) $$\begin{array}{c} \frac{dx}{dt}=x\left(1-\frac{x}{k}\right)-\frac{axy}{1+chx},\\ \frac{dy}{dt}=\frac{bxy}{1+chx}-dy, \end{array}$$

where *x* and *y* represent the density of prey and predator species with carrying capacity *k*. The constant *a* denotes the food intake rate of predator, *b* denotes the food conversion rate to predator, and *d* is the predator's death rate. The constant *c*, *h* being predator's searching rate, and handling time on *x*, respectively. In this model, there is no protection for prey from predator and predator's survival depends on prey alone. Here the predator species *y* totally depends on the prey species *x* and so there is high predation pressure on the prey species. As a result, the prey species has high extinction risk for different searching rate and handling time which is shown in Figure 1. In nature, when the prey population falls below a certain level, the predator searches alternative prey and returns only when the prey population rises to required level. There are large numbers of three or more species food chain system [1, 2] instead of two species system for the survival of prey species. Van Baalen et al. [3] showed the switching fashion from prey species to alternative prey for persistence of predator-prey system. Plants benefit from providing food to predators even when it is also edible to herbivores which is discussed by Van Rijn et al. [4]. Harwood and Obrycki [5] investigated the role of alternative prey in sustaining predator populations. The role of alternative food for biological pest control in predator-prey system is investigated by many scientists [6, 7]. Sahoo [8] studied a food chain model with different functional responses and different growth rates in presence of additional food for construction of real food chain model. Recently, Sahoo [9] showed that additional food is very important for survival of consumer species in an ecosystem. The consequences of providing a predator with additional food and the corresponding effects on the predator-prey dynamics with monotonic and nonmonotonic functional response and its utility in biological control is comparatively studied by Sahoo [10]. But, all of them assumed that the additional food is not dynamic but maintained at a specific constant level either by the nature or by an external agency. In this context, I have proposed a predator-prey model with alternative prey (a dynamic additional food for predator). This model is similar to two prey one predator model.

 The following assumptions are done to formulate the model.

- Let *x* be the prey density, let *y* be the density of alternative prey and the density of the predator is *z*.
- Both preys are distributed uniformly in the habitat.
- The prey and alternative prey grow as per logistic equation in the absence of predators.
- The predator-prey and predator-alternative prey capture rates are of Holling type II.
- The constant *a* is predator's handling time on *x* and *c*, *h* are predator's searching rate and handling time on *y*, respectively.

With the above assumptions, we formulate the following model as

(2) $$\begin{array}{c} \frac{dx}{dt}=x\left(1-\frac{x}{k_{1}}\right)-\frac{pxz}{1+ax+chy},\\ \frac{dy}{dt}=y\left(1-\frac{y}{k_{2}}\right)-\frac{qyz}{1+ax+chy},\\ \frac{dz}{dt}=\frac{\epsilon \left(px+cqy\right)z}{1+ax+chy}-dz, \end{array}$$

where *k*_1_ and *k*_2_ are the carrying capacity of prey (*x*) and alternative prey (*y*), respectively; the constants *p* and *q* are predator's (*z*) food intake rate on prey and alternative prey respectively. The constants *ϵ* and *cϵ* are conversion rates of prey and alternative food to predator, respectively; *d* is constant death rate for predator. 

Here we assume that predator's food intake rate on prey (*x*) is much more greater than that of alternative prey (i.e, *p* ≫ *q*). The parameters *c* and *h* characterize the alternative prey. This formulation implies that the density of prey (*x*) and alternative prey (*y*) are scaled with respect to search rate of the predators, this can be done without loss of generality. The system has to be analyzed with the following initial conditions: *x*(0) ≥ 0, *y*(0) ≥ 0, *z*(0) ≥ 0. 

The main objective of this paper is to investigate the dynamic properties and behaviors of the system. Here I shall analyze the dynamics of the system with respect to predator's (*z*) searching rate *c* and handling time (*h*) on alternative prey *y*. This paper is organized as follows. In Section 2, we show the dissipativeness of the system. The local stability and global stability of the interior equilibrium points of the system are examined in Section 3. Moreover, we discuss the numerical experiment of our system in Section 4. Finally, conclusion is written in Section 5. 

## 2. Theoretical Analysis 

### 2.1. Positive Invariance 

Let *X* = (*x*,*y*,*z*)^*T*^ ∈ *R* ^3^ and

(3) $$\begin{array}{c} F\left(X\right)=\left(\begin{array}{c} F_{1}\left(X\right)\\ F_{2}\left(X\right)\\ F_{3}\left(X\right) \end{array}\right)=\left(\begin{array}{c} x\left(1-\frac{x}{k_{1}}\right)-\frac{pxz}{1+ax+chy}\\ y\left(1-\frac{y}{k_{2}}\right)-\frac{qyz}{1+ax+chy}\\ \frac{\epsilon \left(px+cqy\right)z}{1+ax+chy}-dz \end{array}\right), \end{array}$$

where *F*(*X*) : *C*_+_ → *R* ^3^ and *F* ∈ *C*_+_^*∞*^(*R* ^3^). Then system (2) becomes

(4) $$\begin{array}{c} \dot{X}=F\left(X\right) \end{array}$$

with *X*(0) = *X*_0_ ∈ *R*_+_^3^. It is easy to verify that whenever choosing *X*(0) ∈ *R* ^3^ such that *X*_*i*_ = 0 then [*F*_*i*_(*X*)]_*X*_*i*_=0_ ≥ 0 (for *i* = 1,2, 3). Now any solution of (4) with *X*_0_ ∈ *R*_+_^3^, say *X*(*t*) = *X*(*t*, *X*_0_), is such that *X*(*t*) ∈ *R*_+_^3^ for all *t* > 0 (Nagumo, [11]).

**Theorem 1 .**

All the solutions of the system (2) which initiate in *R*_+_^3^ are uniformly bounded.

**Proof**

Let (*x*(*t*), *y*(*t*), *z*(*t*)) be any solution of the system (2) with positive initial conditions.

Let us consider that

(5) $$\begin{array}{c} w=x+y+z, \end{array}$$

that is,

(6) $$\begin{array}{c} \frac{dw}{dt}=\frac{dx}{dt}+\frac{dy}{dt}+\frac{dz}{dt}. \end{array}$$

Therefore,

(7) dwdt=x(1−xk1)−pxz1+ax+chy+y(1−yk2)−qyz1+ax+chy+ϵ(px+cqy)z1+ax+chy−dz.

Therefore,

(8) $$\begin{array}{c} \frac{dw}{dt}\leq x\left(1-x\right)+y\left(1-y\right)-dz, \end{array}$$

that is,

(9) $$\begin{array}{c} \frac{dw}{dt}\leq 2-\theta \left(x+y+z\right), \end{array}$$

where *θ* = min {1,1, *d*}:

(10) $$\begin{array}{c} \frac{dw}{dt}+\theta w\leq 2. \end{array}$$

Applying the theory of differential inequality we obtain

(11) $$\begin{array}{c} 0<w<\frac{2\left(1-e^{-\theta t}\right)}{\theta }+w\left(x\left(0\right),y\left(0\right),z\left(0\right)\right)e^{-\theta t}. \end{array}$$

For *t* → *∞*, we have 0 < *w* < 2/*θ*.

Hence all the solutions of the system (2) that initiate in *R*_+_^3^ are confined in the region *S* = {(*x*, *y*, *z*) ∈ *R*_+_^3^ : *w* = 2/*θ* + *η*, for any *η* > 0}, which means that all species are uniformly bounded for any initial value in *R*_+_^3^. This proves the theorem.

## 3. Stability Analysis 

### 3.1. Existence and Local Stability of Interior Equilibrium Points

The interior equilibrium point of the system is given by *E* ^*^(*x* ^*^, *y* ^*^, *z* ^*^), where, *y* ^*^ = (*k*_1_ *k*_2_(*p* − *q*) + *k*_2_ *qx* ^*^)/*k*_1_ *p*, *z* ^*^ = (*ϵ*(*k*_1_ − *x*)/*k*_1_ *pd*){((*p* ^2^ *k*_1_ + *cq* ^2^ *k*_2_)*x* + *cqk*_1_ *k*_2_(*p* − *q*))/*k*_1_ *p*}, and *x* ^*^ is the positive root of the equation

(12) $$\begin{array}{c} A{x^{*}}^{2}+Bx^{*}+C=0, \end{array}$$

where, *A* = *ϵ*(*p* ^2^ *k*_1_ + *cq* ^2^ *k*_2_) − *ak*_1_ *pd* − *chk*_2_ *qd*, *B* = (*qk*_1_^2^ *p* − *k*_1_ *p* − *chk*_1_ *k*_2_(*p* − *q*) + *chk*_1_ *k*_2_ *q*)*d* − *ϵk*_1_(*p* ^2^ *k*_1_ + *cq* ^2^ *k*_2_) + *ϵcqk*_1_ *k*_2_(*p* − *q*), and *C* = *k*_1_^2^ *pd* + *dchk*_1_^2^ *k*_2_(*p* − *q*) − *ϵck*_1_^2^ *qk*_2_(*p* − *q*). 

The interior equilibrium point *E* ^*^ exists if *p* > *q*, *k*_1_ > *x* and *B* ^2^ ≥ 4*AC*. 

The Jacobian matrix of the system (2) at the interior equilibrium point *E* ^*^ is

(13) $$\begin{array}{c} J\left(E^{*}\right)=\left(\begin{array}{ccc} A_{11} & A_{12} & A_{13}\\ A_{21} & A_{22} & A_{23}\\ A_{31} & A_{32} & A_{33} \end{array}\right), \end{array}$$

where

(14) A11=1−2x∗k1−p(1+chy∗)z(1+ax∗+chy∗)2,A12=pchx∗z∗(1+ax∗+chy∗)2,A13=−px∗1+ax∗+chy∗,A21=qay∗z∗(1+ax∗+chy∗)2,A22=1−2y∗k2−q(1+ax∗)z(1+ax∗+chy∗)2,A23=−qy∗1+ax∗+chy∗,A31=ϵ[pz∗+c(hp−qa)y∗z∗](1+ax∗+chy∗)2,A32=ϵc[qz∗+(qa−hp)x∗z∗](1+ax∗+chy∗)2,A33=0.

The characteristic equation of the Jacobian matrix *E* ^*^ is given by

(15) $$\begin{array}{c} \lambda^{3}+\Omega_{1}\lambda^{2}+\Omega_{2}\lambda +\Omega_{3}=0, \end{array}$$

where

(16) Ω1=−[A11+A22],Ω2=[A11A22−A12A21−A23A32−A13A31],Ω3=−[A13(A21A32−A22A31)−A23(A11A32−A12A31)].

By the Routh-Hurwitz criteria [12], the positive equilibrium point *E* ^*^(*x* ^*^, *y* ^*^, *z* ^*^) is locally asymptotically stable if and only if Ω_1_ > 0, Ω_3_ > 0, and Ω_1_Ω_2_ − Ω_3_ > 0 hold. 

A sufficient condition for local stability of *E* ^*^(*x* ^*^, *y* ^*^, *z* ^*^) is given by the following theorem.

**Theorem 2 .**

The interior equilibrium point *E* ^*^(*x* ^*^, *y* ^*^, *z* ^*^) for the system (2) is locally asymptotically stable if the following conditions hold:

(17) $$\begin{array}{c} aphx^{*}{z^{*}}^{2}+\epsilon \left[qz^{*}+\left(aq-hp\right)x^{*}z^{*}\right]\left(1+ax^{*}+chy^{*}\right)<0,\\ hqay^{*}{z^{*}}^{2}+\epsilon \left[pz^{*}+\left(ph-aq\right)cy^{*}z^{*}\right]\left(1+ax^{*}+chy^{*}\right)<0,\\ \left(aq+hp\right)+\left(aq-hp\right)\left(ax^{*}-chy^{*}\right)>0. \end{array}$$

**Proof**

From the above derivation, it is clear that *A*_11_ < 0, *A*_12_ > 0, *A*_13_ < 0, *A*_21_ > 0, *A*_22_ < 0, *A*_23_ < 0, *A*_31_ > 0, *A*_32_ > 0, and *A*_33_ = 0. Under these conditions, it is easy to show that Ω_1_ > 0 and Ω_3_ > 0. Now, by calculating Ω_1_Ω_2_ − Ω_3_, we get Ω_1_Ω_2_ − Ω_3_ = (*A*_11_ + *A*_22_)(*A*_23_ *A*_32_ + *A*_13_ *A*_31_ − *A*_11_ *A*_22_) + *A*_11_(*A*_12_ *A*_21_ − *A*_23_ *A*_32_) + *A*_22_(*A*_12_ *A*_21_ − *A*_13_ *A*_31_) + *A*_13_ *A*_21_ *A*_32_ + *A*_23_ *A*_12_ *A*_31_.

Under the conditions (17), it is proven that *A*_12_ *A*_21_ − *A*_23_ *A*_32_ < 0, *A*_12_ *A*_21_ − *A*_13_ *A*_31_ < 0, and *A*_13_ *A*_21_ *A*_32_ + *A*_23_ *A*_12_ *A*_31_ > 0. Therefore, we get Ω_1_Ω_2_ − Ω_3_ > 0. Hence, *E* ^*^(*x* ^*^, *y* ^*^, *z* ^*^) is locally asymptotically stable.

Now, I investigate the global stability of the equilibrium point *E* ^*^ of the system (2). 

### 3.2. Global Stability of Interior Equilibrium Points

**Theorem 3 .**

Suppose that the positive equilibrium point *E* ^*^(*x* ^*^, *y* ^*^, *z* ^*^) is locally asymptotically stable. Then it is a globally asymptotically stable if the following condition holds:

(18) (1+x∗2)2+(1+y∗2)2+dz∗ϵ  <dMϵ−[p(x∗−z∗)−(cz∗−qy∗)]M1+aM+chM+x∗+y∗,

where *M* = 2/*θ*, *θ* = min {1,1, *d*}.

**Proof**

The proof can be done by using a Lyapunov stability theorem which gives a sufficient condition. Now, I consider a positive definite function about *E* ^*^:

(19) $$\begin{array}{c} W\left(x,y,z\right)=W_{1}+W_{2}+\frac{1}{\epsilon }W_{3}, \end{array}$$

where, *W*_1_(*x*, *y*, *z*) = *x* − *x* ^*^ln (*x*/*x* ^*^), *W*_2_(*x*, *y*, *z*) = *y* − *y* ^*^ln (*y*/*y* ^*^), *W*_3_(*x*, *y*, *z*) = *z* − *z* ^*^ln (*z*/*z* ^*^). Therefore,

(20) dWdt=(x−x∗)xdxdt+(y−y∗)ydydt+(z−z∗)z1ϵdzdt,dWdt≤(1−x)(x−x∗)+(1−y)(y−y∗)−d(z−z∗)ϵ   +p(zx∗−xz∗)1+ax+chy−(1−c)qyz1+ax+chy−(cyz∗−qy∗z)1+ax+chy.

Using Theorem 1 without loss of generality, one can assume that there exist a constant *M* = 2/*θ* satisfying *x*(*t*), *y*(*t*), *z*(*t*) < *M*, where *θ* = min {1,1, *d*} and after algebraic calculation we have

(21) dWdt≤−(x−1+x∗2)2−x∗−(y−1+y∗2)2−y∗+(1+x∗2)2+(1+y∗2)2−dϵ(M−z∗)+[p(x∗−z∗)−(cz∗−qy∗)]M1+aM+chM+x∗+y∗.

It is easy to verify that *dW*/*dt* < 0 under the condition (18). Therefore, *W* is a Lyapunov function with respect to *E* ^*^ in the interior positive octant. Hence, the equilibrium point *E* ^*^(*x* ^*^, *y* ^*^, *z* ^*^) is globally asymptotically stable.

## 4. Results and Discussion 

I perform numerical simulations to analyze the dynamics of the proposed model. 

**Figure 2:**
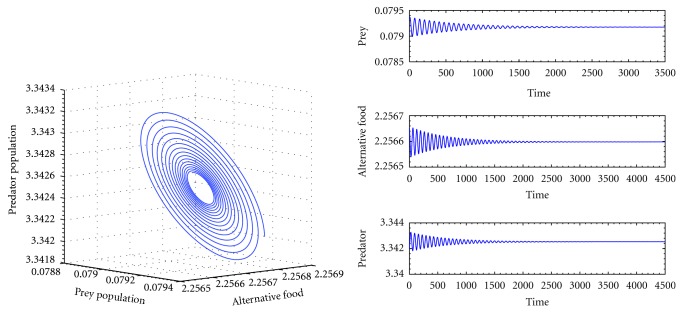
The trajectory and time series diagrams of prey, alternative prey, and predator population of the system for *k*_1_ = 3.0, *k*_2_ = 2.5, *p* = 0.5, *q* = 0.05, *a* = 0.5, *c* = 0.6, *h* = 0.5, *ϵ* = 0.4, and *d* = 0.025.

I choose the parameters as *k*_1_ = 3.0, *k*_2_ = 2.5, *p* = 0.5, *q* = 0.05, *a* = 0.5, *c* = 0.6, *h* = 0.5, *ϵ* = 0.4, and *d* = 0.025. Then it follows from the Theorem 3 that the unique positive interior equilibrium point *E* ^*^(*x* ^*^, *y* ^*^, *z* ^*^) = (0.079135,2.2566,3.34253) is globally stable which is shown in Figure 2. 

**Figure 3:**
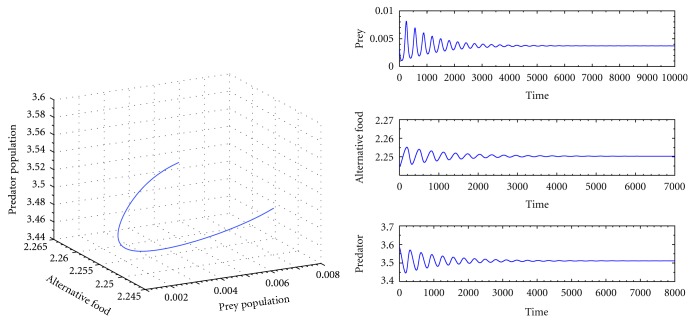
The trajectory and time series diagrams of prey, alternative prey, and predator population of the system for *k*_1_ = 3.0, *k*_2_ = 2.5, *p* = 0.5, *q* = 0.05, *a* = 0.5, *c* = 0.96, *h* = 0.35, *ϵ* = 0.4, and *d* = 0.025.

Next I choose another set of parameters as *k*_1_ = 3.0, *k*_2_ = 2.5, *p* = 0.5, *q* = 0.05, *a* = 0.5, *c* = 0.96, *h* = 0.35, *ϵ* = 0.4, and *d* = 0.025 changing the values of searching rate and handling time. Considering the system (2), with this set of parameters from the Theorem 3 the positive equilibrium point *E* ^*^(*x* ^*^, *y* ^*^, *z* ^*^) = (0.0037155,2.25031,3.51157) exihibits global stability is shown in Figure 3. 

**Figure 4:**
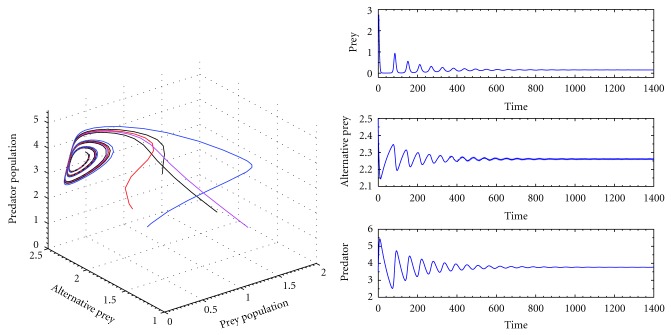
The trajectory and time series diagrams of prey, alternative prey, and predator population of the system for *k*_1_ = 3.0, *k*_2_ = 2.5, *p* = 0.5, *q* = 0.05, *a* = 0.5, *c* = 0.5, *h* = 0.8, *ϵ* = 0.4, and *d* = 0.025.

Again I consider another set of parameters as *k*_1_ = 3.0, *k*_2_ = 2.5, *p* = 0.5, *q* = 0.05, *a* = 0.5, *c* = 0.5, *h* = 0.8, *ϵ* = 0.4, and *d* = 0.025 with change of searching rate and handling time. For this set of values we get the positive interior equilibrium point *E* ^*^(*x* ^*^, *y* ^*^, *z* ^*^) = (0.13333,2.26111,3.76701) is globally stable which is shown in Figure 4. 

From Figures 2, 3, and 4 we observe that the system (2) is globally stable at interior equilibrium point for both low and high handling time as well as searching rate. Therefore our system is globally stable for any handling time and searching rate. 

 Now, I have done the bifurcation analysis of the system with respect to predator's searching rate *c* and handling time *h* taking ecological parameters values *k*_1_ = 3.0, *k*_2_ = 2.5, *p* = 0.5, *q* = 0.05 < *p*, *a* = 0.5, *ϵ* = 0.4, and *d* = 0.025 which is fixed throughout the bifurcation analysis. 

**Figure 5:**
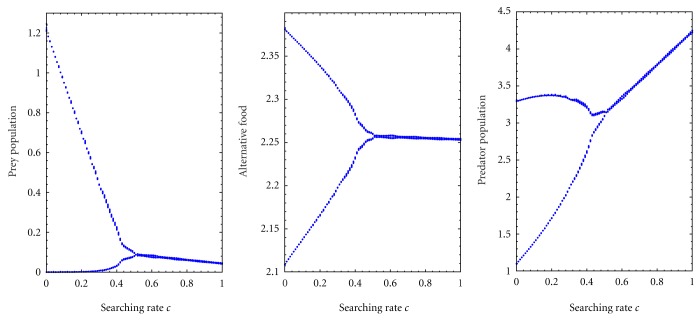
Bifurcation diagram of the system with respect to searching rate *c* with fixed handling time *h* = 0.5 and for *k*_1_ = 3.0, *k*_2_ = 2.5, *p* = 0.5, *q* = 0.05, *a* = 0.5, *ϵ* = 0.4, and *d* = 0.025. It depicts periodic and stable dynamics.

Figure 5 is the bifurcation diagram of the system with respect to searching rate *c* with fixed handling time *h* = 0.5. From Figure 5, I observe that prey population has extinction risk for lower values of searching rate *c* and for higher searching rate it reaches steady state. On the other hand, alternative prey and predator population show periodic behaviour for lower searching rate *c* and for higher searching rate they go to steady states. Therefore, alternative prey and predator population have no extinction risk; they survive in the system always. 

**Figure 6:**
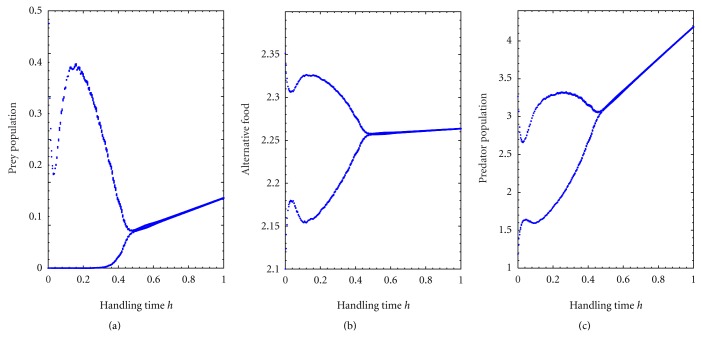
Bifurcation diagram of the system with respect to handling time *h* with fixed searching rate *c* = 0.5 and for *k*_1_ = 3.0, *k*_2_ = 2.5, *p* = 0.5, *q* = 0.05, *a* = 0.5, *ϵ* = 0.4, and *d* = 0.025. It depicts periodic and stable dynamics.

**Figure 7:**
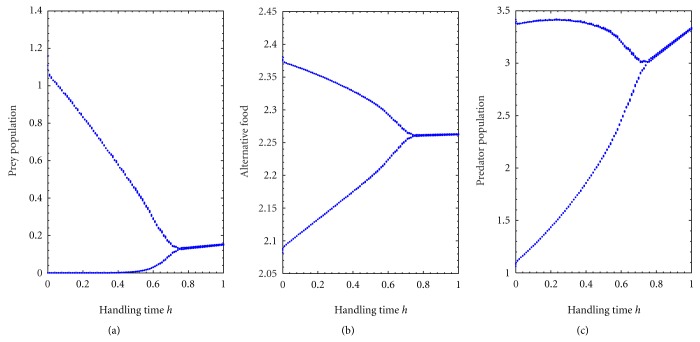
Bifurcation diagram of the system with respect to handling time *h* with fixed searching rate *c* = 0.8 and for *k*_1_ = 3.0, *k*_2_ = 2.5, *p* = 0.5, *q* = 0.05, *a* = 0.5, *ϵ* = 0.4, and *d* = 0.025. It depicts periodic and stable dynamics.

Bifurcation analysis with respect to handling time *h* for fixed searching rate *c* = 0.5, is shown in Figure 6. From Figure 6, I observe that prey population extinct for low values of handling time *h* but for high values of handling time *h* the system (2) settles down to steady state. Alternative prey and predator population have no extinction risk, they survive in the system always. For higher searching rate at *c* = 0.8, the Figure 7 shows that the prey population extinction risk increases for higher values of handling time *h*. Therefore, the increase of searching rate *c* shows prey's extinction from the system for higher values of handling time compare to low searching rate. 

## 5. Conclusions 

I have proposed a predator-prey model in presence of alternative prey. I have derived the condition of local asymptotic stability and global stability of the interior equilibrium points. Theoretically, I have shown the global stability under certain condition. Numerically, I have done bifurcation analysis of the system with respect to predator's searching rate *c* and handling time *h*. From bifurcation diagrams we observe that the system's dynamics are either periodic or stable. The periodic behaviour of the system indicates the existence of stability of the system. From bifurcation analysis, I can conclude that when searching rate is very low, the prey populations are easily captured by predators, and, therefore, prey population has high extinction risk while the alternative prey has no extinction risk. Also it is observed that the prey populations will survive in the system if the searching rate of predator is very high. Similar dynamics are shown with respect to handling time-taking fixed-searching rate. For higher predator's handling time, prey population, and alternative prey go to steady state. Therefore, I can conclude that the predator population never extinct for presence of alternative prey.
